# Maize leaves salt-responsive genes revealed by comparative transcriptome of salt-tolerant and salt-sensitive cultivars during the seedling stage

**DOI:** 10.7717/peerj.19268

**Published:** 2025-04-10

**Authors:** Mingfang Ji, Sirui Xu, Zhongxian Ma, Chengnan Xiao, Jiangting Xu, Yanfang Zhu, Ronghao Cai, Chen Bo

**Affiliations:** 1 Anhui Provincial Engineering Laboratory for Efficient Utilization of Featured Resource Plants, College of Life Sciences, Huaibei Normal University, Huaibei, China; 2 National Engineering Laboratory of Crop Stress Resistance Breeding, School of Life Sciences, Anhui Agricultural University, Hefei, China

**Keywords:** Maize seedling leaves, Salt stress, RNA sequencing, Salt tolerance, Differentially expressed genes

## Abstract

Maize (*Zea mays*) is a crop of significant global importance, yet its productivity is considerably hindered by salt stress. In this study, we investigated two maize cultivars, one exhibiting high salt tolerance (ST) and the other showing salt sensitivity (SS) at the seedling stage. The ST cultivar demonstrated superior seedling survival rates, higher relative water content, and lower electrolyte leakage and malondialdehyde levels in its leaves after both 3-day and 7-day salt treatments, when compared to the SS cultivar. To explore the molecular basis of these differences, we performed comparative transcriptome sequencing under varying salt treatment durations. A total of 980 differentially expressed genes (DEGs) were identified. Gene ontology (GO) functional enrichment analysis of DEGs indicated that the oxidation-reduction process, phosphorylation, plasma membrane, transferase activity, metal ion binding, kinase activity, protein kinase activity and oxidoreductase activity process is deeply involved in the response of ST and SS maize varieties to salt stress. Further analysis highlighted differences in the regulatory patterns of transcription factors encoded by the DEGs between the ST and SS cultivars. Notably, transcription factor families such as AP2/ERF, bZIP, MYB, and WRKY were found to play crucial roles in the salt stress regulatory network of maize. These findings provide valuable insights into the molecular mechanisms underlying salt stress tolerance in maize seedlings.

## Introduction

Over one-third of the world’s irrigated agricultural land is currently affected by salinization, which is a major abiotic stressor that limits crop production (Zhao et al., 2020). Salt stress induces a broad array of physiological, biochemical, and molecular changes within plants, including the regulation of salt uptake by roots, the enhancement of osmolyte accumulation, the modulation of reactive oxygen species (ROS) levels, and the alteration of gene and transcription factors (TFs) expression associated with salt stress responses (Cheeseman, 2013; Li & Liu, 2019; Munns, 2005). Over time, higher plants have evolved intricate physiological and biochemical mechanisms to mitigate the detrimental effects of high salinity, a result of long-term survival and evolutionary adaptation (Yang & Guo, 2018; Zhu, 2016). Investigating the key genes and molecular pathways involved in plant salt tolerance, alongside the genetic engineering approaches aimed at enhancing salt tolerance, have emerged as a focal point in crop molecular breeding and the genetic improvement of stress resistance.

Maize (*Zea mays*) is one of the world’s most important food crops and a raw material for livestock feed and industrial processing. Recent research has identified several salt-responsive genes and regulators in maize that enhance plant salt tolerance (She et al., 2024; Wang et al., 2025). For better salt tolerance, ZmHKT1 (an HKT-type Na^+^-selective transporter protein) and ZmHAK4 (a membrane-localized Na^+^-selective transporter protein) were found to work together to promote shoot Na^+^ rejection and keep the Na^+^/K^+^ balance (Zhang et al., 2018a; Zhang et al., 2019). The radicle of germinating embryos mainly expresses another gene, ZmHAK17, which encodes a Na^+^ transporter located in the plasma membrane. Under salt stress, the increased transcript level of *ZmHAK17* promoted the exocytosis of Na^+^ from the radicle, thus preventing the accumulation of Na^+^ in the embryo and reducing the effect of salt stress on germination (Wang et al., 2024). Under salt stress, the protein level of ZmRR1 (an A-type response regulator) decreased. Its inhibition of ZmHP2, a positive regulator of cytokinin signaling, was deregulated, followed by ZmHP2-mediated cytokinin signaling up-regulating the expression of *ZmMATE29* (encoding a tonoplast-located Cl^−^ transporter), which in turn upregulated the expression of *ZmMATE29* by promoting Cl^−^ exclusion from shoots by compartmentalizing Cl^−^ into the vacuoles of root cortex cells and improving salt stress tolerance in transgenic maize (Yin et al., 2023). A NAM, ATAF1/2, and CUC2 (NAC) transcription factor, ZmNAC84 was found to respond to various abiotic stresses. Overexpression of *ZmNAC84* reduces H_2_O_2_ accumulation and enhances resistance to plant salt stress by increasing catalase (CAT) activity. Further studies showed that ZmNAC84 improves salt stress tolerance by binding to specific regions of the promoter of the downstream gene *ZmCAT1* in maize seedlings (Pan et al., 2024). In addition, it was found that moderate expression of the sterilizing gene *ORF355* in mitochondria increased intracellular nicotinamide adenine dinucleotide (NAD) content and altered intracellular metabolic homeostasis, activating the antioxidant defense system, thus improving salt tolerance in S-type cytoplasmic male sterility (CMS-S) maize (Xiao et al., 2022).

Except for studying one or a few genes associated with salt stress, large-scale transcriptomics analyses can shed more complete light on the molecular mechanisms of salt tolerance in maize. The researchers used 348 natural populations with wide genetic variation to conduct genome-wide association studies for 27 traits, including shoot length, root length, shoot fresh weight and salt tolerance index of seedlings germinated for 10 days under normal and salt stress conditions, and identified 12 salt tolerance candidate genes by combining with transcriptome differential expression analysis (Luo et al., 2021). Further studies revealed that the CRISPR/Cas9 edited mutant of the *ZmCLCg* and the ethyl methylsulfone (EMS) mutant of the *ZmPMP3* had significantly lower root length, root fresh weight, aboveground length, and aboveground fresh weight than that of the wild-type (WT) maize plants under salt stress conditions, confirming that the candidate genes, *ZmCLCg* and *ZmPMP3*, are associated with salt tolerance in maize (Luo et al., 2021). Additional studies have identified an AP2/ERF-like transcription factor, ZmEREB57, using a salt-treated comparative transcriptome induced by various abiotic stresses in maize. ZmEREB57 affects the accumulation of endogenous oxo phytodienoic acid (OPDA) and jasmonate (JA) by regulating the expression of *ZmAOC2*, which is involved in the regulation of phytohormone signaling, redox, and the expression of a variety of genes encoding genes related to salt stress response (Zhu et al., 2023b). Comprehensive physiological and comparative transcriptomic analyses of maize seedlings subjected to salt stress were conducted utilizing the paternal (cmh15) and maternal (CM37) progenitors of the maize cultivar An’nong 876. The findings revealed that the responses of cmh15 and CM37 to salt stress were significantly associated with photosynthetic activity and redox processes. Furthermore, notable differences were observed in the regulatory patterns of hormone signaling pathways and the TFs encoded by the differentially expressed genes (GEDs) (Zhang et al., 2022). In summary, transcriptomic analysis has elucidated alterations in key genes and pathways associated with the stress response in maize, thereby providing insights into the mechanisms underlying stress perception and response. However, in previous studies, most maize salt-treated comparative transcriptome sequencing was based on a specific treatment time. To obtain candidate genes for further genetic manipulation, using a set of maize varieties with opposite phenotypes and dynamically analyzing their accumulation levels of DEGs under different salt treatment times is preferable for obtaining critical salt-responsive genes to provide targets for molecular design breeding (Zhu et al., 2023a).

In this study, we aimed to elucidate the mechanisms underlying maize responses to salt stress using transcriptomic approaches. For obtaining salt stress-responsive genes and further elucidating the response mechanism of maize to salt stress, we selected two cultivars, salt-tolerant (ST) and salt-sensitive (SS) with significant differences in salt-treated phenotypes, and compared the expression profiles of DEGs and analyzed the functions of significant salt stress-related genes. The results of the study deepen the understanding of the salt response mechanism of maize and provide a theoretical basis for the selection and breeding of new salt-tolerant varieties of maize.

## Materials & Methods

### Plant materials and salt stress treatment

The experimental materials were two maize cultivars, salt-tolerant CM1 and salt-sensitive HG12. Seeds were provided by the National Engineering Laboratory of Crop Stress Resistance Breeding, Anhui Agricultural University, P.R. China. The seeds were carefully chosen and then grown in a greenhouse with a temperature regime of 28 °C in the light phase and 23 °C in the dark phase, adhering to a 16-hour light and 8-hour dark cycle. Upon reaching the three-leaf developmental stage, the seedlings were subjected to irrigation with a 300 mM sodium chloride (NaCl) solution daily for a duration of one week. In contrast, the seedlings designated as the control group received standard irrigation practices. The third leaves were collected from control and salt-stressed treated maize after 3 d and 7 d of treatment, respectively. Each had three biological replicates for RNA-Seq experiments, then frozen in liquid nitrogen immediately and stored at −80 °C.

### Protocol of DAB and NBT staining and measurement of physiological and biochemical indexes

Leaves of both maize cultivars were immersed in a solution containing 1 mg/mL of 3,3′-Diaminobenzidine (DAB) and 0.5 mg/mL of nitroblue tetrazolium (NBT), followed by vacuum infiltration for 20 min. The samples were then incubated in the dark for 8 h at 28 °C and boiled for 5 min in a solution of ethanol: lactic acid: glycerin (3:1:1) before being observed (Chen et al., 2019). The relative water content (RWC), relative electrolyte leakage (REL), and hydrogen peroxide (H_2_O_2_) levels of both control and salt-treated seedlings were quantified according to previously established protocols (Bo et al., 2022). Malondialdehyde (MDA) content, along with the activities of superoxide dismutase (SOD) and peroxidase (POD), expressed as fresh weight (FW), were assessed using commercial assay kits (Nanjing Jiancheng Bioengineering Institute, China). Each experiment was conducted with three replications.

### cDNA library construction and sequencing

TRIzol (Invitrogen, Carlsbad, CA, USA) was utilized to extract total RNA from the third leaves. Agarose gel electrophoresis was used to evaluate the purity and concentration of the RNA, and the NanoDrop 2500 spectrophotometer (Thermo Fisher Scientific, Wilmington, DE, USA) was used to quantify the results. Oligo(dT)-coated magnetic beads were used to enrich the mRNA, and then a fragmentation buffer was used to create random RNA fragments. Using random hexamer primers and short RNA fragments as templates, the first strand of complementary DNA (cDNA) was created. The second strand of cDNA was then created using deoxy-ribonucleoside triphosphate (dSNTPs), Ribonuclease H (RNase H), and DNA polymerase I. After using magnetic beads to purify the resultant double-stranded cDNA, an end repair process was carried out, which involved appending a single adenine (A) nucleotide to the 3′ends. The cDNA was then ligated to sequencing adapters. Polymerase chain reaction (PCR) amplification was used to select and enrich the appropriate fragments. Using an Agilent 2100 Bioanalyzer (Agilent Technologies, Santa Clara, CA, USA), the purity and concentration of the RNA were further confirmed. To create strand-specific RNA-seq libraries, equal quantities of RNA from each sample were combined and sequenced at LC Biotechnology Co., Ltd. (Hangzhou, China) using an Illumina NovaSeq™ 6000 platform.

### Data processing

Data were collected as previously described in Bo et al. (2024). The raw data generated by the sequencer were referred to as raw reads. These raw reads from each sample were subjected to quality filtering, which involved the removal of reads containing more than 10% ambiguous nucleotides, low-quality reads with more than 40% bases having a *Q* value ≤ 20, and adapter sequences, following the Illumina adapter guidelines. Cutadapt software (v1.9) was used to remove the reads that contained adaptor contamination (Martin, 2011). After removing the low-quality bases and undetermined bases, we used the HISAT2 software (v2-2.0.4) to map reads to the maize genome (*Zea mays*, Zm-B73-REFERENCE-GRAMENE-4.0) (Kim, Langmead & Salzberg, 2015). Finally, all transcriptomes from all samples were merged to reconstruct a comprehensive transcriptome using the gffcompare software (v0.9.8) (Pertea & Pertea, 2020).

### Gene annotation and DEG’s GO and KEGG enrichment

The differentially expressed mRNAs were selected with log_2_fold change > 1 and *P* value < 0.05 by R package edgeR (Robinson, McCarthy & Smyth, 2010). StringTie and ballgown were used to estimate the expression levels of all transcripts and perform expression level for mRNAs by calculating fragments per kilobase of transcript per million mapped reads (FPKM) (Pertea et al., 2015). GO enrichment (GOseq v1.34.1) and KEGG web service (http://www.kegg.jp/, accessed on November 21, 2022) were performed on the edgeR-derived DEGs, with significance defined as false discovery rate (FDR) < 0.05 (Young et al., 2010). The PlantTFDB 5.0 database (https://planttfdb.gao-lab.org/, accessed on 14 September 2024) was used to find TFs within the significant gene sets.

### Validation of RNA-seq data by qRT-PCR

The qRT-PCR experiment was analyzed according to previously described methods (Bo et al., 2024). A total of eight DEGs were chosen for qRT-PCR validation. The Evo MMLV RT Premix for qRT-PCR (Accurate Biotechnology Co., Ltd., Changsha, China) was used to create single-stranded complementary DNA (cDNA) in accordance with the manufacturer’s instructions. Primer3Plus (http://www.primer3plus.com/, accessed on 14 September 2024) was used to build gene-specific primers. The PCR reactions were conducted using the FastStart Essential DNA Green Master (Roche, Basel, Switzerland). The process and reaction system were the same as those reported in previous research (Bo et al., 2024; Xue et al., 2024). Glyceraldehyde -3-phosphate dehydrogenase (GAPDH) was used as an internal control for normalization (Zhang et al., 2021a), with three technical replicates for each cDNA sample. The primer sequences used in the qRT-PCR assays are provided in Table S1. The 2^−ΔΔCT^ technique was used to calculate relative gene expression levels (Livak & Schmittgen, 2001).

### Statistical analysis

GraphPad Prism 7.0 and SPSS 22.0 software (SPSS Inc., Chicago, IL, USA) were used for statistics analysis. Samples were analyzed in biological triplicate, and the data are presented as mean ± standard deviation. Statistical significance was determined using two-way _ANOVA_ and Student’s *t*-test (*P < 0.05, **P < 0.01). Bars with different letters indicate statistically significant differences in means (*P* < 0.05).

## Results

### Phenotypic responses of two maize cultivars to salt stress

**Figure 1:**
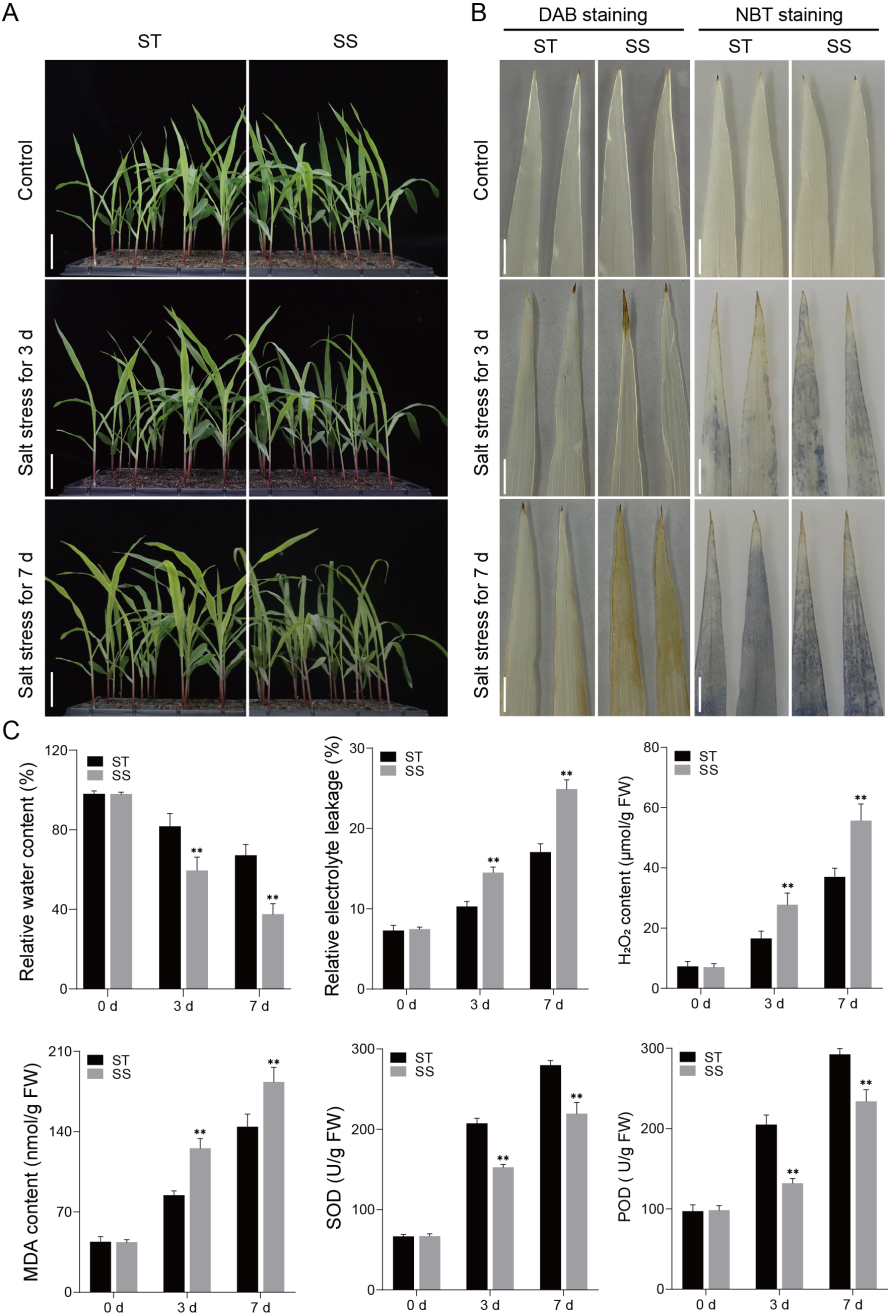
Salt treatment phenotypic characterization of the ST and SS maize cultivars. (A) Phenotypes of the ST and SS seedings to salt stress. Plants were grown to the four-leaf stage in soil and then irrigated with saline water containing 300 mM NaCl for 0, 3, and 7 days. The scale bars are six cm. (B) DAB and NBT staining of the third leaves of ST and SS seedings treated with 300 mM NaCl for 0, 3 and 7 days. The scale bars are one cm. (C) The RWC, REL, H_2_O_2_ and MDA content, SOD and POD activities of ST and SS seedings treated with 300 mM NaCl for 0, 3 and 7. Data means ± SDs (*n* = 3). The symbols * and ** indicate significant differences between ST and SS seedings at *P* < 0.05 and *P* < 0.01, respectively.

Salt-tolerant (ST) and salt-sensitive (SS) seedling varieties were subjected to a 300 mM NaCl treatment, and their phenotypic responses to salt stress were assessed once the third leaf of the seedlings reached full expansion. Under control conditions, both SS and ST seedlings grew well. However, after 3 and 7 days of salt treatment, the SS seedlings exhibited significant wilting and dieback, while the ST seedlings showed much less phenotypic damage (Fig. 1A). DAB and NBT staining revealed that both SS and ST leaves displayed staining marks after salt treatment, indicative of ROS production. However, SS leaves showed significantly deeper staining compared to ST leaves, with the difference becoming more pronounced at 7 days of treatment (Fig. 1B). Further studies showed that there were no differences in physiological indices such as RWC, REL and MDA content between SS and ST plants before treatment. When plants were exposed to salt stress for 3 days and 7 days, ST plants still had high levels of RWC, but their levels of REL and MDA were much lower than those of SS plants (Fig. 1C). Additionally, analysis of ROS-related enzyme activities revealed that the activities of SOD and POD were significantly higher in ST plants compared to SS plants at both 3 and 7 days of salt treatment (Fig. 1C). The findings suggest that ST exhibited a greater capacity for tolerance to salt stress in comparison to SS plants.

### RNA-seq analysis and identification of DEGs

**Figure 2:**
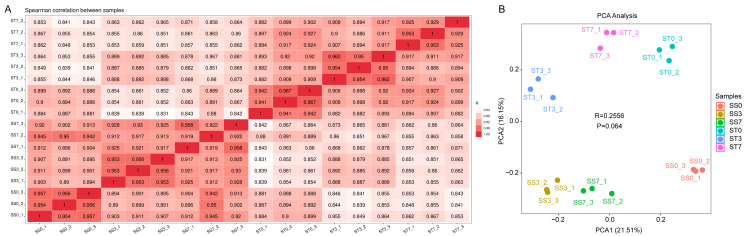
Sample-sample correlation heatmap and PCA analysis plot of the differentially expressed genes (DEGs) in three comparisons of ST and SS seedings treated with 300 mM NaCl for 0, 3 and 7 days. (A) Sample-sample correlation heatmap of the 18 samples. (B) PCA analysis plot.

To identify genes responsive to salt stress, transcriptome analyses were conducted on maize leaves treated with 300 mM NaCl for 0, 3, and 7 days. A total of eighteen samples were analyzed: SS0/ST0 (0-day treatment), SS3/ST3 (3-day treatment), and SS7/ST7 (7-day treatment), with three replicates for each group. After quality filtering, the total number of reads and bases per library ranged from 35.85 million to 55.75 million and 5.38 G to 8.36 G, respectively. The Q30 and guanine-cytosine (GC) contents ranged from 97.22% to 98.14% and from 54.50% to 57.00%, respectively, reflecting high-quality sequencing data (Table 1). Gene expression levels were normalized using the fragments per kilobase per million reads (FPKM) method. Sample correlation heatmaps and principal component analysis (PCA) showed high reproducibility of gene expression data across the biological replicates, with the samples being evenly distributed along the axes (Fig. 2).

**Table 1:** Summary of the sequence data from Illumina sequencing.

Sample	Raw data read	Base	Valid data read	Base	Valid ratio (reads)	Q20%	Q30%	GC content%
SS0_1	4,2613,860	6.39G	3,9415,284	5.91G	92.49	99.89	97.61	56.50
SS0_2	4,3119,214	6.47G	4,0005,472	6.00G	92.78	99.90	97.55	56.50
SS0_3	4,3846,674	6.58G	4,0656,334	6.10G	92.72	99.91	97.71	55.50
SS3_1	4,7281,604	7.09G	4,4370,978	6.66G	93.84	99.87	97.22	56
SS3_2	3,5848,870	5.38G	3,3462,010	5.02G	93.34	99.89	97.66	56
SS3_3	4,6606,360	6.99G	4,3272,608	6.49G	92.85	99.89	97.87	56
SS7_1	4,8037,638	7.21G	4,5431,432	6.81G	94.57	99.92	97.45	54.50
SS7_2	5,0894,370	7.63G	4,7769,600	7.17G	93.86	99.92	97.87	56.50
SS7_3	4,8748,334	7.31G	4,6130,382	6.92G	94.63	99.93	98.14	55
ST0_1	5,2810,244	7.92G	4,7926,954	7.19G	90.75	99.89	97.47	55
ST0_2	5,4401,302	8.16G	5,1438,552	7.72G	94.55	99.92	97.79	56
ST0_3	5,0981,496	7.65G	4,8129,494	7.22G	94.41	99.88	97.60	57
ST3_1	5,2564,124	7.88G	4,8849,238	7.33G	92.93	99.87	97.74	56
ST3_2	5,3032,132	7.95G	4,9577,982	7.44G	93.49	99.86	97.43	55
ST3_3	5,5754,280	8.36G	5,1898,358	7.78G	93.08	99.87	97.40	56
ST7_1	5,1332,990	7.70G	4,8377,582	7.26G	94.24	99.89	97.80	57.50
ST7_2	4,3328,076	6.50G	4,0874,050	6.13G	94.34	99.89	97.51	57.50
ST7_3	5,1187,182	7.68G	4,8123,066	7.22G	94.01	99.90	97.67	56.50

**Figure 3:**
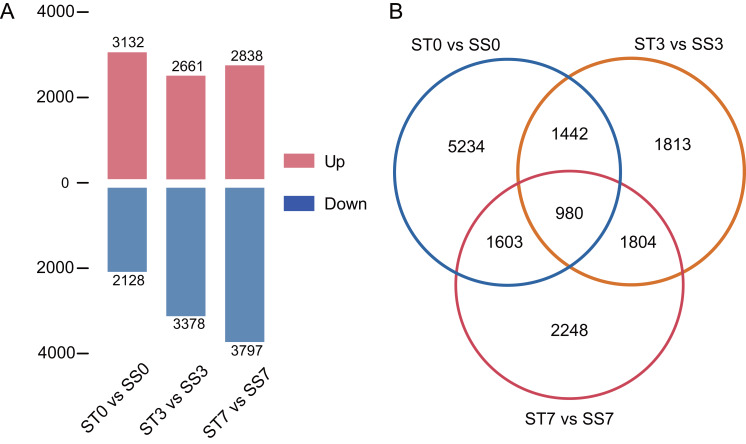
Numbers of DEGs in ST0 *vs.* SS0, ST3 *vs.* SS3, and ST7 *vs.* SS7 comparisons (A) and overlap between DEGs (B).

Genes were considered significantly differentially expressed (DEGs) when the fold change was ≥ 2 and the adjusted *P* value was ≤ 0.05. In the ST0 *vs.* SS0 comparison, we identified 3,132 up-regulated and 2,128 down-regulated genes. In the ST3 *vs.* SS3 comparison, we identified 2,661 up-regulated and 3,378 down-regulated genes. And in the ST7 *vs.* SS7 comparison, we identified 2,838 up-regulated and 3,797 down-regulated genes (Fig. 3A, Table S2). The Venn statistics of the comparative analysis between the groups identified 980 DEGs that were common to salt stress response (Fig. 3B). The specific expression levels and details of these DEGs are shown in Table S3.

### Functional classification of DEGs

**Figure 4:**
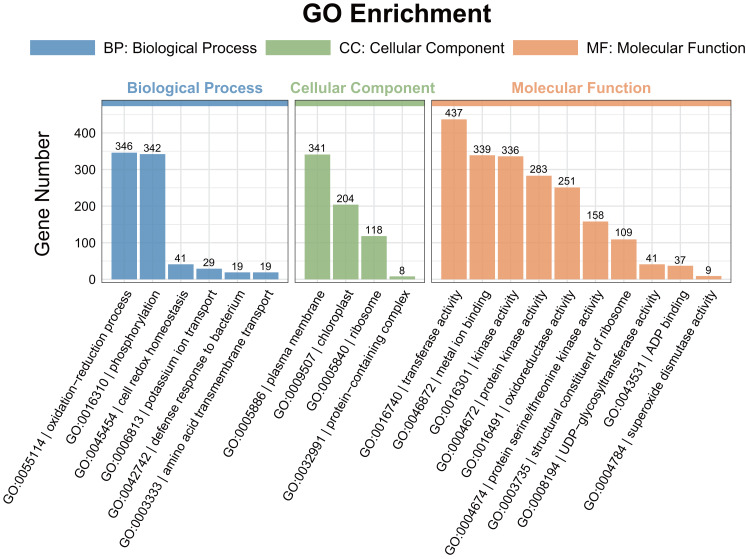
Gene ontology (GO) enrichment analysis of the DEGs detected in ST0 *vs.* SS0, ST3 *vs.* SS3, and ST7 *vs.* SS7 comparisons. The topmost enriched GO terms under the three main GO categories are shown.

A gene ontology (GO) enrichment analysis was conducted to explore the functional roles of the differentially expressed genes (DEGs). Among the DEGs identified in the pairwise comparisons, 15 shared GO terms with a *Q*-value of < 0.05 were found in the biological process category (Fig. 4). The significantly enriched biological processes (BPs) included the response to oxidation–reduction processes (GO:0055114) and phosphorylation (GO:0016310). The most enriched cellular components (CCs) were associated with the plasma membrane (GO:0005886). In the molecular function (MF) category, transferase activity (GO:0016740), metal ion binding (GO:0046872), kinase activity (GO:0016301), protein kinase activity (GO:0004672), and oxidoreductase activity (GO:0016491) were significantly enriched.

**Figure 5:**
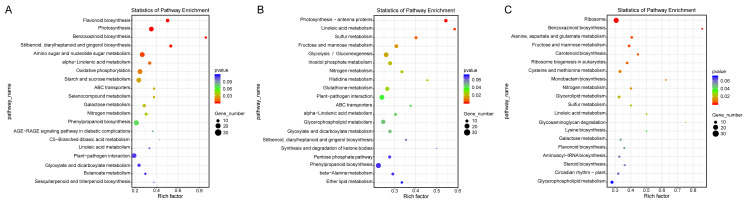
KEGG enrichment map of DEGs in ST0 *vs.* SS0 (A), ST3 *vs.* SS3 (B), and ST7 *vs.* SS7 (C) comparisons.

Additionally, a pathway-based analysis using Kyoto Encyclopedia of Genes and Genomes (KEGG) pathway enrichment revealed that the most enriched pathways across the three comparisons were ribosome (ko03010), photosynthesis (ko00195), amino sugar and nucleotide sugar metabolism (ko00520), and oxidative phosphorylation (ko00190) (Fig. 5). These findings provide insights into the biological functions and potential regulatory mechanisms of DEGs in response to salt stress.

### Analysis of salt stress-responsive TFs

**Figure 6:**
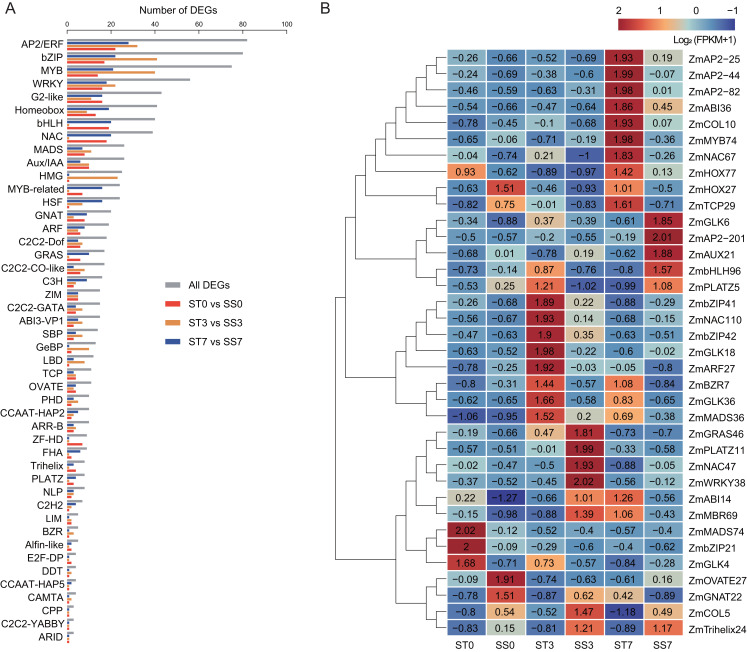
Transcription factors (TFs) encoded by DEGs detected in ST0 *vs.* SS0, ST3 *vs.* SS3, and ST7 *vs.* SS7 comparisons. (A) Classification and statistics of the TF families. (B) Heatmaps of the FPKM+1 values of the 36 TFs.

To predict salt stress-responsive TFs in this study, DEGs in response to NaCl treatment in the two maize strains were annotated using the PlantTFDB database. A total of 980 DEGs encoding TFs were identified, representing 46 different TF families (Fig. 6A). Among these, 13 TF families accounted for over 64% of the salt stress-responsive TFs, including the AP2/ERF, bZIP, MYB, WRKY, G2-like, Homeobox, bHLH, NAC, MADS, Aux/IAA, HMG, MYB-related, and HSF families. The AP2/ERF family was the largest, containing 82 DEGs, with 22, 32, and 28 DEGs in the 0, 3, and 7-day comparisons, respectively. The bZIP family had 80 DEGs, with 17, 41, and 22 DEGs in the three comparisons, respectively. In addition, 75 MYB TFs, 56 WRKY TFs, and 43 G2-like TFs were identified as differentially expressed.

Further analysis revealed that 36 common TFs were differentially expressed across all three comparison groups, with 20 up-regulated and 16 down-regulated (Fig. 6B, Table 2). Among the common TFs, *ZmbZIP21* has been reported as salt stress-related candidate gene (Hu et al., 2023), and *ZmWRKY38* (also referred as *ZmWRKY20*) has been confirmed to be involved in the salt stress response (Bo et al., 2022). There are some other genes, such as *ZmAP2-25* (Zhang et al., 2024), *ZmAP2-82* (Zhang et al., 2021b), *ZmAUX21* (Zhang et al., 2018b), *ZmGLK4* (Wang et al., 2020), *ZmNAC47* (Tian et al., 2023), and *ZmNAC110* (Li et al., 2021), have been implicated in various abiotic stress responses, including salt stress, in previous studies. These TFs may serve as key regulators in the salt stress response of maize.

**Table 2:** Stress-related TFs between ST and SS seedings.

	Gene name	Gene ID	Fold increase/decrease		
			ST0 *vs.* SS0	ST3 *vs.* SS3	ST7 *vs.* SS7
Up	ZmABI14	Zm00001d049369	2.77	26.73	3.01
	ZmABI36	Zm00001d049094	4.08	6.79	6.05
	ZmAP2-201	Zm00001d021892	4.59	33.78	7.16
	ZmAP2-25	Zm00001d036298	4.17	6.33	4.03
	ZmAP2-44	Zm00001d029884	8.20	4.02	18.58
	ZmAP2-82	Zm00001d019116	2.72	0.03	0.04
	ZmbZIP21	Zm00001d044546	4.22	3.29	2.69
	ZmbZIP41	Zm00001d034447	2.39	6.25	2.87
	ZmbZIP42	Zm00001d040500	16.87	77.17	22.41
	ZmGLK36	Zm00001d002439	4.20	2.93	7.68
	ZmGLK4	Zm00001d023402	12.13	19.76	84.76
	ZmGLK6	Zm00001d032190	7.42	2.84	9.87
	ZmGRAS46	Zm00001d044065	7.56	9.77	2.14
	ZmHOX77	Zm00001d045398	4.92	3.22	2.55
	ZmMADS74	Zm00001d044899	5.7	2.55	2.24
	ZmMBR69	Zm00001d045581	2.09	2.54	4.43
	ZmNAC110	Zm00001d024268	2.41	2.35	2.17
	ZmNAC47	Zm00001d008817	2.01	2.04	2.79
	ZmNAC67	Zm00001d023669	2.26	3.00	2.12
	ZmWRKY38	Zm00001d005622	4.45	2.67	3.89
Down	ZmARF27	Zm00001d045026	0.2	0.47	0.17
	ZmAUX21	Zm00001d013302	0.16	0.01	0.15
	ZmbHLH96	Zm00001d007382	0.17	5.11	0.07
	ZmBZR7	Zm00001d006677	0.24	0.10	0.02
	ZmCOL10	Zm00001d037327	0.43	4.66	0.01
	ZmCOL5	Zm00001d017885	0.26	2.37	0.12
	ZmGLK18	Zm00001d015226	0.09	0.36	0.07
	ZmGNAT22	Zm00001d013596	0.14	0.05	0.03
	ZmHOX27	Zm00001d015671	0.13	0.10	0.16
	ZmMADS36	Zm00001d043589	0.06	0.31	0.05
	ZmMYB74	Zm00001d012544	0.09	0.16	0.04
	ZmOVATE27	Zm00001d038284	0.26	0.28	0.05
	ZmPLATZ11	Zm00001d017682	0.31	0.48	0.12
	ZmPLATZ5	Zm00001d002489	0.40	0.23	0.21
	ZmTCP29	Zm00001d012725	0.09	0.14	0.07
	ZmTrihelix24	Zm00001d016876	0.18	0.27	0.13

### Confirmation of RNA-seq data by real-time quantitative PCR

**Figure 7:**
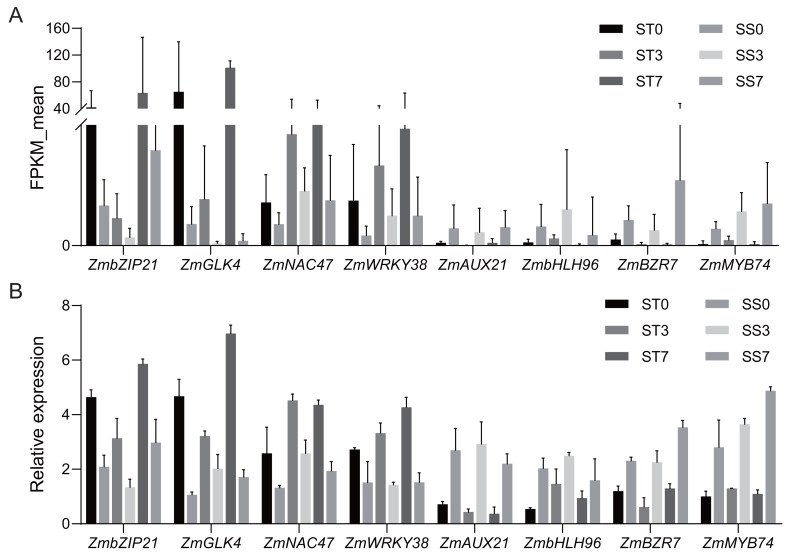
Quantitative real-time PCR validations of DEGs characterized by RNA-seq. (A) Average FPKM values acquired by RNA-seq. (B) The relative expression levels of candidate genes by qRT-PCR.

Based on the functional annotation and analysis results, along with previously published research, eight candidate genes (four up-regulated and four down-regulated) from Table 2 were selected for qRT-PCR validation. The same RNA samples used for the RNA sequencing (RNA-Seq) analysis were utilized for the qRT-PCR analysis. For up-regulated genes, the qRT-PCR results revealed a similar magnitude of fold changes as seen in RNA-Seq, which indicates that the changes in gene expression due to salt stress were reliably detected. For the down-regulated genes, the qRT-PCR analysis confirmed a consistent reduction in expression, further validating the down-regulation patterns identified in RNA-Seq (Fig. 7). These results indicate that the expression profiles by qRT-PCR were highly consistent with the trends of FPKM values obtained from RNA-Seq, confirming the reliability of RNA-Seq data. The correlation coefficient (R-squared) between the two methods was 0.85, which confirms that the RNA-Seq data were reliable and the expression trends observed were accurate (Fig. S1). This consistency between RNA-Seq and qRT-PCR results validates the findings and supports the reliability of the identified DEGs in response to salt stress.

## Discussion

Salt stress is a major abiotic factor that limits maize yield, and understanding the genetic mechanisms underlying salt tolerance is critical for developing salt-resistant maize varieties (Van Zelm, Zhang & Testerink, 2020; Wu et al., 2020). Previous studies on salt tolerance in maize have typically focused on a single growth period of a single variety, without considering the effect of multiple growth stages or comparing different varieties (Zhang et al., 2022). To address this limitation, our study employed two maize cultivars with distinctly contrasting phenotypes under salt stress. We sequenced their transcriptomes at multiple time points of salt treatment, enabling the identification of genes closely associated with the salt stress response across different growth periods. This approach provides a more comprehensive understanding of the dynamic gene expression changes that occur during salt stress and could facilitate the development of more salt-tolerant maize cultivars by targeting genes that exhibit consistent differential expression across multiple time points and varieties.

After 3 and 7 days of 300 mM NaCl treatment, transcriptome sequencing and differential gene expression analysis identified approximately 18,000 DEGs, which was consistent with our expected results. A Venn diagram showed that the three comparison groups shared 980 DEGs, which suggests that there is a core group of genes involved in the response to salt stress. This suggests that even at the seedling stage, maize has developed diverse and robust mechanisms to respond to abiotic stress, highlighting the complexity and adaptability of its stress response pathways.

A large number of studies have shown that salt stress leads to metabolic imbalance and increased ROS production, which leads to oxidative stress that damage lipids, DNA and proteins (Jiang et al., 2007). In this study, it was found that REL, H_2_O_2_ and MDA contents, and SOD and POD activities increased over time in both maize cultivars under 300 mM NaCl treatment conditions for 3 and 7 days. However, the antioxidant enzyme activities in the ST cultivar were consistently higher than those in the SS cultivar, suggesting that the ST plants have a more efficient defense mechanism, particularly during the early stages of salt stress (Fig. 1). The ability to remove ROS and H_2_O_2_ is essential for plant stress tolerance. Key pathways involved in ROS scavenging include the ascorbate-glutathione cycle, glutathione peroxidase, catalase, and peroxiredoxin/thioredoxin systems (Gill & Tuteja, 2010). In this study, the GO term associated with the oxidation–reduction process had the largest number of DEGs, with a total of 346. This significant difference in the expression of genes involved in redox reactions may help explain the observed differences in salt stress tolerance between the two maize cultivars (Fig. 4). The greater expression of antioxidant-related genes in the ST cultivar could contribute to its superior ability to mitigate oxidative damage and maintain cellular integrity under salt stress.

Protein modification is crucial in regulating a wide range of cellular functions, including binding, catalysis, regulation, and altering physical properties. Phosphorylation, one of the most prevalent post-translational modifications (PTMs), can transiently change protein properties, such as enzymatic activity, subcellular localization, structure, stability, and interactions with other proteins (Vu et al., 2016). Notably, protein phosphorylation is central to transmitting stress signals from the cell surface to the nucleus, acting as a universal biochemical signal regulating stress response. It is also a key PTM in abscisic acid (ABA) signaling, which is involved in various stress responses, including salt stress (Xu & Zhang, 2015). In the BP category, the GO term related to phosphorylation ranked second after oxidation–reduction processes, with 342 DEGs identified. This highlights the importance of phosphorylation in regulating cellular responses to stress. Previous studies have recognized phospholipid signaling pathways, which are regulated by protein phosphorylation, as being crucial in plant responses to environmental stresses, including salt stress (Sui et al., 2008). The prominence of phosphorylation-related genes in this study suggests that phosphorylation plays a significant role in the salt stress response, potentially modulating key pathways that enhance stress tolerance in maize.

The KEGG analysis revealed that several pathways, including photosynthesis, nucleotide sugar metabolism, and oxidative phosphorylation, were significantly enriched with specific DEGs (Fig. 5). These findings suggest that these pathways play a critical role in modulating salt stress resistance in maize, either by repressing or activating key responses. This aligns with previous studies, highlighting the importance of these pathways in plant stress tolerance (Zhang et al., 2022). The differential enrichment of these pathways between ST and SS maize varieties further indicates that variations in salt tolerance may be attributed to differences in how these pathways are regulated. Such differences could influence the ability of maize to manage oxidative stress, energy production, and metabolic adjustments under salt stress, ultimately contributing to the observed phenotypic variation in stress tolerance.

In our study, a total of 36 differentially expressed TFs were identified, including key members from the AP2/ERF, bZIP, MYB, WRKY, and other families. These TFs have been well-documented in previous research for their critical roles in regulating both biotic and abiotic stress tolerance, as well as plant defense responses (Ding et al., 2021; Feng et al., 2020; Li et al., 2018; Liu et al., 2024). Among the 36 differentially expressed TFs, 20 were up-regulated and 18 were down-regulated, suggesting a complex regulatory network in response to salt stress. One key TFs identified was *ZmWRKY38* (also known as *ZmWRKY20*), a salt stress-responsive gene previously reported in EMS-induced maize mutants (Bo et al., 2022). ZmWRKY20 interacts with ZmWRKY115 in the nucleus and synergistically represses its expression by directly binding to the *ZmbZIP111* promoter, increasing the sensitivity of maize seedlings to salt stress. This further proves the intricate network of TFs involved in salt stress regulation. Another notable gene, *ZmbZIP21*, was up-regulated under salt treatment, consistent with previous reports (Hu et al., 2023), suggesting its involvement in salt stress response. Additionally, ZmNAC47 and ZmNAC110, both members of the maize NAC transcription factor family, have previously been shown to be involved in drought stress (Li et al., 2021; Tian et al., 2023). In our study, these genes were up-regulated in response to salt stress, indicating that they may participate in the regulation of multiple abiotic stress pathways. Furthermore, several TFs identified in this study have also been implicated in biotic stress and other maize growth and development aspects. For instance, ZmGLK36 promotes resistance to rice black-streaked dwarf virus by enhancing JA biosynthesis and JA-mediated defense responses (Xu et al., 2023). *ZmMYB74* and *ZmMYB138* are regulated by zma-miR159 and are involved in regulating maize grain size and weight (Wang et al., 2023). Interestingly, *ZmMYB74* was found to be induced by salt stress in this study, suggesting that they may also play a role in abiotic stress responses. These findings underscore the multifaceted roles of TFs in regulating maize’s response to both biotic and abiotic stresses, and while these candidate genes show potential, further functional studies are needed to fully confirm their biological roles in salt stress tolerance and broader stress regulatory networks.

To verify the reliability of the candidate genes, we finally conducted a correlation analysis. The correlation coefficient (R-squared) between RNA-Seq and qRT-PCR expression levels was 0.85, indicating a strong positive relationship between the two datasets (Fig. S1). This high correlation not only confirms the reliability of the RNA-Seq data but also highlights the rigor of the experimental design in capturing accurate gene expression profiles. Furthermore, among the selected genes, those showing significant up-regulation, such as *ZmWRKY38*, are known to play a key role in the salt stress response by regulating ROS (Bo et al., 2022). Conversely, the down-regulated genes, such as *ZmMYB74*, are associated with pathways that might be suppressed under salt stress conditions to redirect energy and resources toward stress adaptation. The qRT-PCR results emphasize the dynamic regulation of key genes during salt stress, further validating their roles in stress tolerance mechanisms. These findings underscore the validity of the DEGs identified in this study and provide additional support for their involvement in the response to salt stress.

## Conclusions

This study compared the transcriptomic responses of two maize cultivars, the salt-tolerant CM1 and the salt-sensitive HG12, after 3- and 7-day salt treatments at the three-leaf stage. The experiment involved six groups and 18 samples, using an RNA-seq approach to investigate the DEG patterns in response to salt stress. We identified significant alterations in gene expression related to oxidative phosphorylation, photosynthesis, and nucleotide sugar metabolism, which are crucial for the energy management and metabolic flexibility required under salt stress conditions. Notably, several transcription factor families, such as AP2/ERF, bZIP, MYB, and WRKY, emerged as significantly correlated with salt tolerance, underscoring their crucial roles in modulating the stress response of maize. The expression patterns of some transcription factors known to be involved in salt stress responses, such as ZmbZIP21 and ZmWRKY38, highlight their potential roles as key regulatory nodes. Furthermore, the validation of RNA-Seq data through qRT-PCR has confirmed the reliability of our transcriptomic insights, enhancing the confidence in these results. The findings present critical reference data for advancing the understanding of genetic mechanisms governing salt stress tolerance in maize during the seedling stage.

## Supplemental Information

10.7717/peerj.19268/supp-1 Supplemental Information 1 Correlation analysis between the RNA-seq and qRT-PCR results

10.7717/peerj.19268/supp-2 Supplemental Information 2 Primers used in this study

10.7717/peerj.19268/supp-3 Supplemental Information 3 RNA-seq results for ST and ST maize cultivars used in the study

10.7717/peerj.19268/supp-4 Supplemental Information 4 The specific expression levels and details of DEGs

10.7717/peerj.19268/supp-5 Supplemental Information 5 Raw data for the plant experiments (Figures 1C and 7B)

## Additional Information and Declarations

### Data Availability

The following information was supplied regarding data availability: The raw sequence data are available in the NCBI Sequence Read Archive: PRJNA1174350. The raw data are available in the Supplemental Files.
